# Supplementary material for: Validation of the Multidimensional Sociosexual Orientation Inventory (SOI-M) in the Chilean population
Source: PLoS One. 2025 Aug 14;20(8):e0329036. doi: 10.1371/journal.pone.0329036 (PMC12352763; doi:10.1371/journal.pone.0329036)
Supplement: S1 Table — (DOCX) [file pone.0329036.s001.docx]

Los ítems se responden en una escala Likert de 7 puntos, siendo 1 = totalmente en desacuerdo y 7 = totalmente de acuerdo. Los ítems 13, 14 y 15 son preguntas abiertas de respuesta numérica.

|  | 1 | 2 | 3 | 4 | 5 | 6 | 7 |
| --- | --- | --- | --- | --- | --- | --- | --- |
| 1. Puedo imaginarme fácilmente estando cómodo/a y disfrutando del sexo casual con diferentes mujeres/hombres |  |  |  |  |  |  |  |
| 2. Puedo imaginarme disfrutando de un encuentro sexual breve con una mujer/hombre que me resulta muy atractiva/o. |  |  |  |  |  |  |  |
| 3. Puedo imaginarme fácilmente disfrutando de un encuentro sexual de una noche con una mujer/hombre que nunca más vuelva a ver |  |  |  |  |  |  |  |
| 4. El sexo sin amor está bien. |  |  |  |  |  |  |  |
| 5. Consideraría tener relaciones sexuales con una extraña/o si se me garantiza que es seguro y la mujer/hombre fuese atractiva/o. |  |  |  |  |  |  |  |
| 6. Creo que debo tomar las oportunidades sexuales cuando las encuentro |  |  |  |  |  |  |  |
| 7. Tendría que estar profundamente vinculado con una mujer/hombre (emocional y Psicológicamente) antes de sentirme cómodo y disfrutando del sexo con ella/él. |  |  |  |  |  |  |  |
| 8. Estoy interesado en mantener una relación romántica a largo plazo con una mujer/hombre especial. |  |  |  |  |  |  |  |
| 9. Espero tener una relación romántica que perdure el resto de mi vida. |  |  |  |  |  |  |  |
| 10. Las relaciones románticas a largo plazo no son para mí. |  |  |  |  |  |  |  |
| 11. Para mí no es importante encontrar una pareja romántica a largo plazo. |  |  |  |  |  |  |  |
| 12. Me puedo imaginar estableciéndome románticamente con una mujer/hombre especial. |  |  |  |  |  |  |  |
| 13. Durante toda tu vida, ¿con cuántas personas distintas has tenido relaciones sexuales? |  | | | | | | |
| 14. Durante el último año, ¿con cuántas personas distintas has tenido relaciones sexuales? |  | | | | | | |
| 15. ¿Con cuántas personas has tenido relaciones sexuales en una sola ocasión? |  | | | | | | |
